# Early Physiotherapy of Post‐Surgical Scars Using a Topical Cannabidiol (VeroDol CBD) Cream Gel: Three Case Reports

**DOI:** 10.1111/jocd.70775

**Published:** 2026-02-26

**Authors:** Davide Coucourde, Dionisio Franco Barattini

**Affiliations:** ^1^ Studio Coucourde Torino Italy; ^2^ Clinical Research Consultant Genova Italy

**Keywords:** cosmetic, elasticity, keloids, mobility, physiotherapy, surgical scar, TECAR therapy

To the Editor,

Physiotherapy for surgical scars includes massage, soft tissue mobilization, and myofascial techniques, supported by systematic reviews and controlled studies showing improvements in pain, adhesions, thickness, and mobility [1, 2]. In particular, adhesions beneath scars can delay or prevent early manual intervention.

Cannabidiol and β‐caryophyllene, which have been investigated for their potential anti‐inflammatory properties [3], are included in a commercially available cosmetic cream gel together with capsaicin, arnica, devil's claw, escin, menthol, and camphor. We report three cases of post‐surgical scar adhesion management observed during routine physiotherapy practice using this product, VeroDol CBD cream gel (Tuscopharm, Italy).

**Case 1**

A 24‐year‐old man underwent Latarjet surgery for recurrent anterior shoulder dislocation. Physiotherapy started 7 days post‐surgery, coinciding with staple removal, twice weekly for 4 weeks. Sessions consisted of 60‐min skin rolling (pincé‐roulé), preceded by application of approximately 5 mL of the tested cream gel to the scar. Pain on pressure (VAS 0–10) improved from 8 at baseline to 0 at Day 28; tissue mobility (Likert scale: 1 = normal, 10 = worst) improved from 8 to 1. At Day 28, the surgeon confirmed complete recovery without limitation of range of motion (ROM).

**Case 2**

A 72‐year‐old woman began physiotherapy 45 days after reverse shoulder replacement. For 3 weeks, she received weekly sessions combining scar frictions, traction, joint mobilization, and application of the tested cream gel (~5 mL per session). VAS pain decreased from 7 to 1, while tissue mobility improved from 5 to 1. At Day 21, both the surgeon and physiotherapist confirmed complete clinical recovery (Figure 1).

**FIGURE 1:**
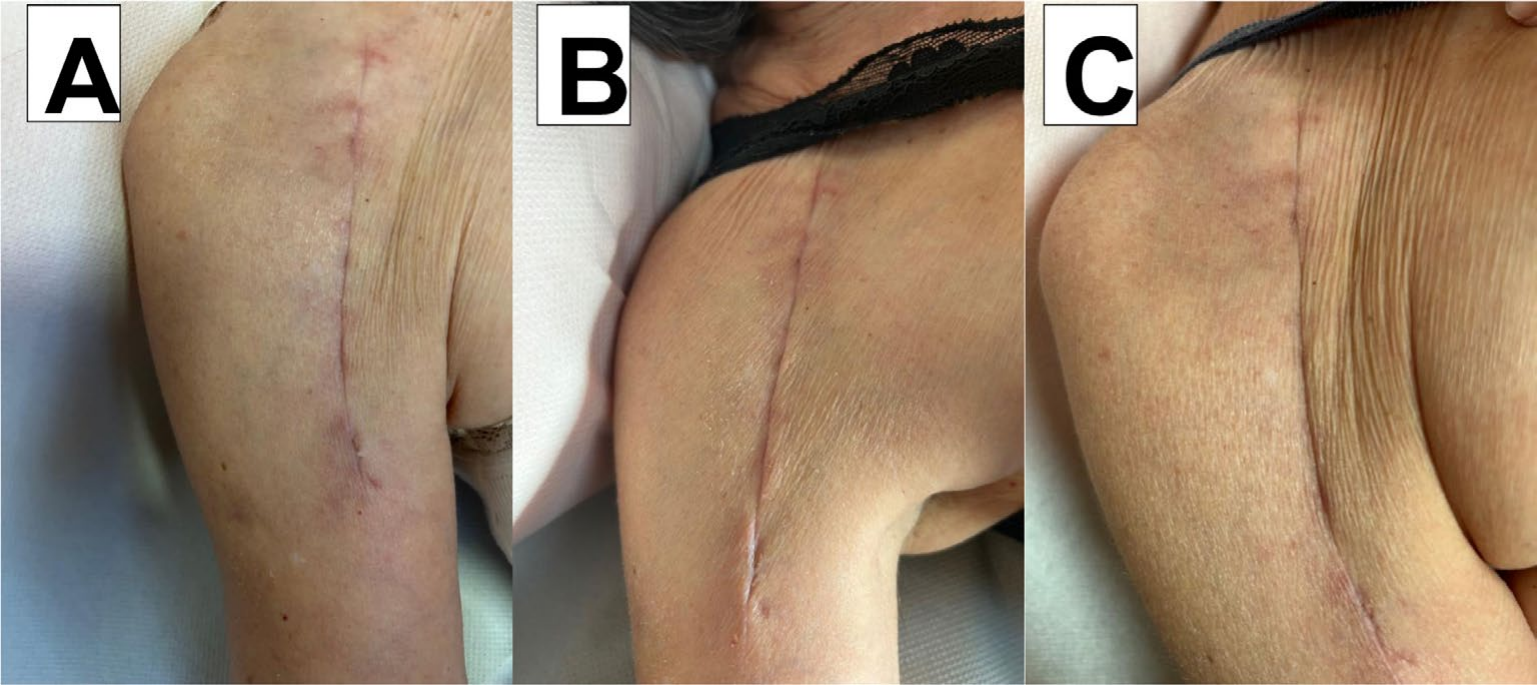
Clinical appearance of the scar following reverse shoulder replacement: (A) before treatment (baseline); (B) on Day 7 of treatment; and (C) on Day 21 of treatment (last day). The surgeon evaluates the scar's recovery clinically.

**Case 3**

A 19‐year‐old man with a keloid scar following scaphoid osteosynthesis presented with raised tissue, redness, and pain on touch, making initial skin rolling impossible. Three sessions (baseline, Day 7, and Day 21) of Transferred Energy Capacitive and Resistive (TECAR) therapy were performed to reduce pain and improve scar tissue mobility and elasticity [4]. During TECAR, the conductive cream was mixed with 30% of the tested cream gel. The patient also self‐applied approximately 5 mL of the tested cream gel twice daily from baseline to Day 21. VAS pain decreased from 9 to 3, and mobility improved from 10 to 3. Progressive changes in scar pigmentation and perilesional skin appearance were observed, and by Day 21 the scar became treatable with skin rolling (Figure 2).

**FIGURE 2:**
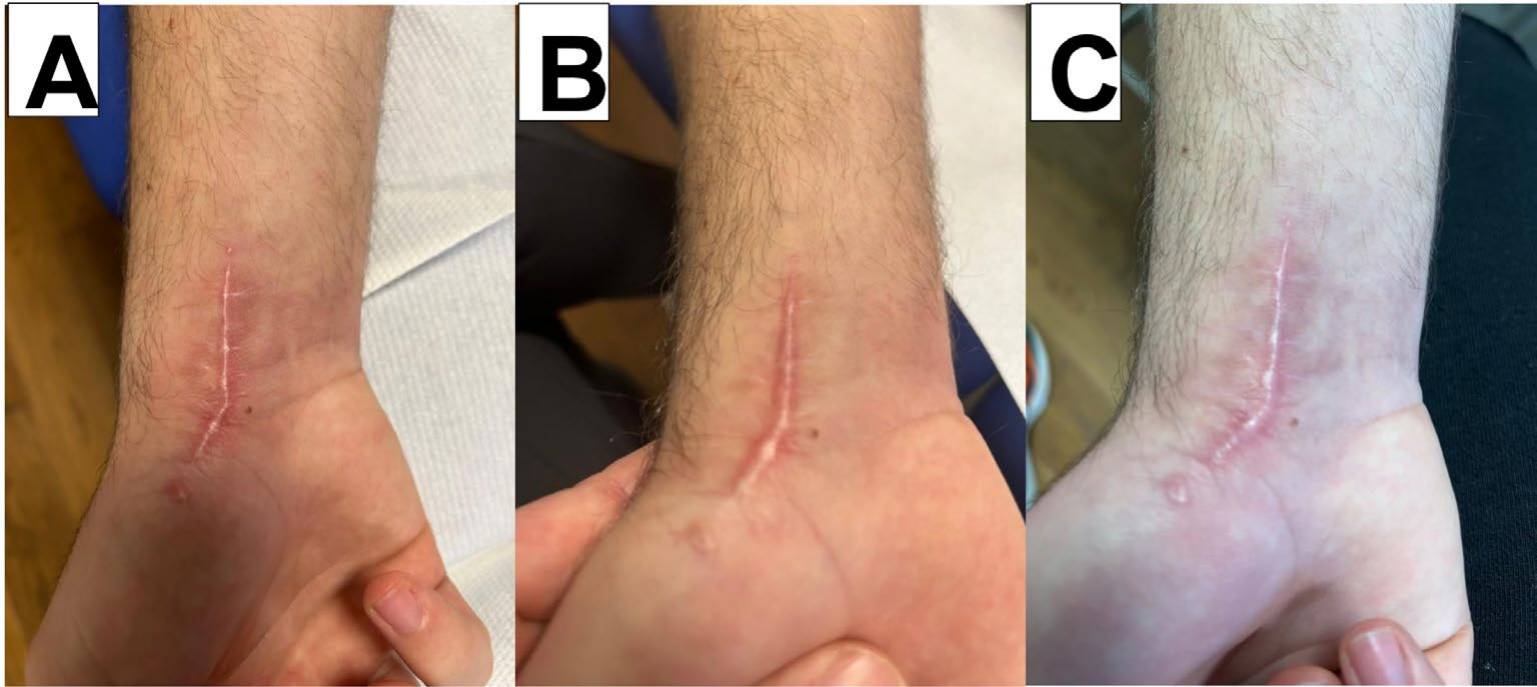
Clinical appearance of a keloid scar following scaphoid osteosynthesis. Three sessions of Transferred Energy Capacitive and Resistive (TECAR) therapy were administered: one at baseline (A), one on Day 7 (B), and one on Day 21 (C). The conductive cream was mixed with 30% tested cream gel. Additionally, the patient self‐administered the cream gel alone twice daily from baseline to Day 21. On Day 21, the scar was treatable with skin rolling. TECAR therapy uses high‐frequency currents, applied via electrodes, to stimulate ions and tissues either superficially or deeply. This treatment generates endogenous heat, which reduces pain and inflammation. It also improves tissue repair by increasing microcirculation, cellular metabolism, and lymphatic drainage.

These small case observations suggest that integrating the topical application of the tested cream gel into physiotherapy protocols was associated with reduced pain and improved tissue mobility during treatment sessions. No comorbidities, smoking, concomitant medications able to affect wound healing, or adverse events were reported. In Case 1, early intervention was feasible immediately after staple removal. In Case 2, recovery was achieved despite delayed initiation of physiotherapy. In Case 3, the combination of the tested cream gel and TECAR therapy appeared to improve scar conditions sufficiently to allow manual treatment within three weeks.

Limitations include the small sample size, lack of statistical analysis, absence of blinded assessment, and restricted outcome measures [5]. The Likert scale used to assess tissue mobility has not been formally validated; therefore, findings should be interpreted as exploratory. Nevertheless, subjective scales, clinician agreement, and the practical “treatable/not treatable” threshold for skin rolling and accompanying aesthetic improvement supported the observed clinical changes.

This preliminary experience is based on uncontrolled case observations; therefore, larger controlled and randomized clinical trials [6] are required to better characterize the potential role of topical cosmetic formulations as adjuncts to physiotherapy in post‐surgical scar management. Integration with other modalities, such as laser therapy, ultrasound, and radiofrequency should also be evaluated.

## Author Contributions

D.C. conceived and supervised the study; D.C. and D.F.B. analyzed data; D.C. and D.F.B. wrote the manuscript; D.F.B. made manuscript revisions. All authors reviewed the results and approved the final version of the manuscript.

## Funding

The authors have nothing to report.

## Disclosure

Tuscopharm had no role in the collection of the three case reports described in the manuscript, or in the analyses, or interpretation of data, or in the writing of the manuscript, or in the decision to publish the results.

## Ethics Statement

The authors confirm that the ethical policies of the *Journal of Cosmetic Dermatology*, as noted on the journal's author guidelines page, have been adhered to. According to Italian regulations no ethical approval was required as the used product was a cosmetic and its administration was performed by the physiotherapist following his power of care and after the signature of informed consent by each patient.

## Consent

The patients gave their consent for the publication of the case details and anonymized photos.

## Conflicts of Interest

The authors declare no conflicts of interest. D.F.B. is an independent clinical research consultant and D.C. is a private physiotherapist.

## Data Availability

The data that support the findings of this study are available from the corresponding author upon reasonable request.
